# Organic covalent modification to improve thermoelectric properties of TaS_2_

**DOI:** 10.1038/s41467-022-32058-w

**Published:** 2022-07-29

**Authors:** Shaozhi Wang, Xiao Yang, Lingxiang Hou, Xueping Cui, Xinghua Zheng, Jian Zheng

**Affiliations:** 1 Beijing National Laboratory for Molecular Sciences, Key Laboratory of Organic Solids, Institute of Chemistry, Chinese Academy of Sciences, Beijing, 100190 China; 2 University of Chinese Academy of Sciences, Beijing, 100049 China; 3 Institute of Engineering Thermophysics, Chinese Academy of Sciences, Beijing, 100190 China

**Keywords:** Two-dimensional materials, Electronic properties and devices

## Abstract

Organic semiconductors are attracting considerable attention as a new thermoelectric material because of their molecular diversity, non-toxicity and easy processing. The side chains which are introduced into two-dimensional (2D) transition metal dichalcogenides (TMDs) by covalent modification lead to a significant decrease in their thermal conductivity. Here, we describe a simple approach to preparing the side chains covalent modification TaS_2_ (SCCM-TaS_2_) organic/inorganic hybrid structures, which is a homogeneous and non-destructive technique that does not depend on defects and boundaries. Electrical conductivity of 3,401 S cm^−1^ and a power factor of 0.34 mW m^−1^ K^−2^ are obtained for a hybrid material of SCCM-TaS_2_, with an in-plane thermal conductivity of 4.0 W m^−1^ K^−1^, which is 7 times smaller than the thermal conductivity of the pristine TaS_2_ crystal. The power factor and low thermal conductivity contribute to a thermoelectric figure of merit (ZT) of ~0.04 at 443 K.

## Abstract (web summary)

The improvement of thermoelectric performance of materials by reducing lattice thermal conductivity is challenging. Here, the authors find the side chains covalent modification of transition metal disulfides reducing the lattice thermal conductivity.

## Introduction

Thermoelectric (TE) devices can directly convert heat to electricity without moving parts or working fluids^1^, which provide an environmentally friendly route for power generation or refrigeration, and have thus attracted significant attention. Generally, the thermoelectric energy conversion efficiency is determined by the thermoelectric figure of merit (ZT), defined as ZT = S^2^σT/κ, where S, σ and T represent the seebeck coefficient, electrical conductivity, absolute temperature respectively, and κ is the thermal conductivity that includes contributions from both phonons and electrons^2,3^. The optimization of ZT is severely limited by the anti-correlation between those transport coefficients. Therefore, it is desirable for a TE material to have favorable electrical conductivity and low thermal conductivity^4,5^. Organic semiconductors offer numerous advantages for thermoelectric applications, such as molecular diversity, large-area preparation, high flexibility, low weight, easy processing, material abundance and non-toxicity^6,7^. Organic compounds rely on *sp*^2^ hybrid carbon-carbon bonds to conduct electricity, and the light weight of carbon atoms is conducive to effective phonon transport, which makes them theoretically have higher electrical conductivity and thermal conductivity. But in fact, the thermal conductivity of organic compounds is relatively low, and the side chain is one of the main factors causing this phenomenon^8–10^.

Two-dimensional (2D) transition metal dichalcogenides (TMDs) have attracted considerable interest owing to their respectable carrier mobility, density of states and tunable bandgap^11^. Although the electrical conductivity of TMDs material is very high, it is not suitable for thermoelectric applications because of its high thermal conductivity. If the side chains can be introduced into the interlayers of TMDs materials by organic covalent modification, we can reduce the thermal conductivity and obtain a satisfactory ZT value. It is very difficult to introduce enough side chains in the interlayer of TMDs materials. Most of the previous methods relied on boundaries or defects modification^12^, and only a few methods can introduce substituents in the basal plane^13,14^. These methods need to exfoliate the bulk single crystal into single layers for full reaction, which will damage the original large single crystal structure, produce small fragments and form grain boundaries in the crystal, thereby greatly reducing the carrier mobility. Therefore, it is of great significance to intercalate and covalently modify the inner layer of TMDs while maintaining its single crystal structure.

Here, we demonstrate a simple covalent modification strategy that does not depend on boundaries or defects. The single crystal used in the experiment is 2H-TaS_2_. The organic groups (tert-Butyl isocyanate) are covalently bonded to 2D TMDs single crystal (TaS_2_) directly to form the side chains covalent modification organic/inorganic hybrid structure, referred to as SCCM-TaS_2_. The method can be used to conduct organic covalent modification of layered bulk single crystals deep into the crystal lattices. It is a homogeneous and non-destructive covalent modification method, which has no boundaries and defects formation. The achieved organic/inorganic hybrid structures have good stability. The thermal conductivity was found to be significantly suppressed owing to the side chains effect, leading to a significant improvement of ZT at 443 K. In general, the organic hybrid technology with side chains can effectively enhance the phonon scattering and improve the ZT value of TMDs material. This strategy can be extended to other layered materials, such as Bi_2_Se_3_^15^, TiS_2_^16^ and SnSe_2_^17^.

## Results and discussion

### Preparation of SCCM-TaS_2_

**Fig. 1:**
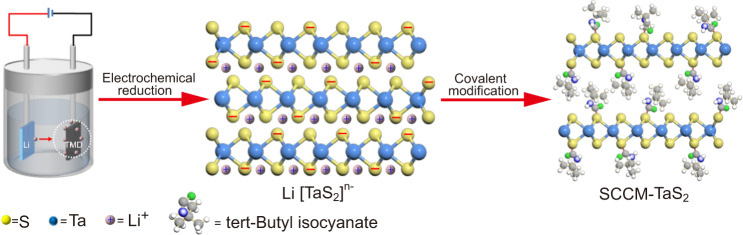
Schematic of synthesis processes of SCCM-TaS_2_. Schematic of the electrochemical reaction and side chains covalent modification for SCCM-TaS_2_ organic/inorganic hybrid structure.

In this article, we conduct organic side chains covalent modification of 2H-TaS_2_ crystals by reacting with isocyanates (Fig. 1). The TaS_2_ crystals are difficult to react with other substances to form covalent bonds, because there are no dangling bonds and all chemical bonds are saturated in the TaS_2_ crystals. Therefore, the TaS_2_ crystals do not react with isocyanates directly. The electrochemical reduction was used to charge the TaS_2_ crystals and activate the crystal lamellae. Additionally, the electron doping effect was used to increase the electron cloud density of sulfur atoms, which can enhance the nucleophilic ability, and make it easy for sulfur atoms to attack the carbon atom of isocyanates to form covalent bonds. The SCCM-TaS_2_ hybrid structure was achieved by immersing the electrochemical reduction product in tert-Butyl isocyanate at room temperature.

### Structural characterization

**Fig. 2:**
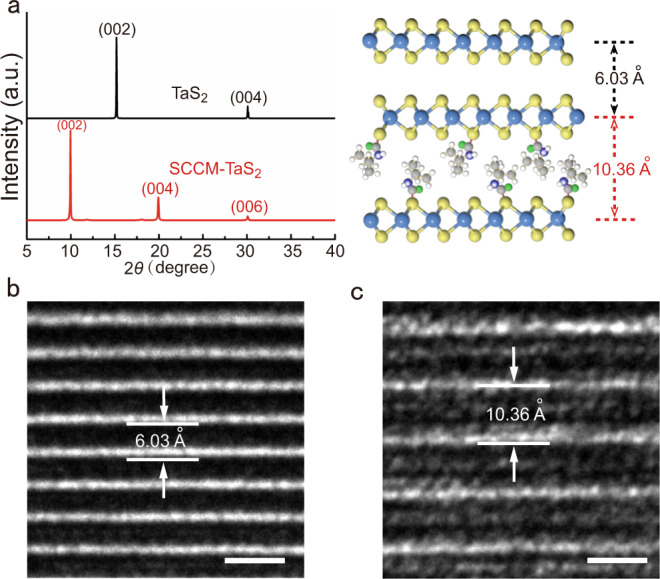
Structural characterization from the pristine TaS_2_ and SCCM-TaS_2_ hybrid structure. **a** XRD pattern and schematic of pristine TaS_2_ single crystal and SCCM-TaS_2_ hybrid structure. TEM characterization of TaS_2_ single crystal (**b**) and SCCM-TaS_2_ hybrid structure (**c**). (Scale bars, 1 nm).

After organic side chains covalent modification, the interlayer distance of TaS_2_ expanded obviously. Studies with X-ray diffraction (XRD) showed a 67% increase in the interlayer distance from TaS_2_ to SCCM-TaS_2_ hybrid structure (Fig. 2a). In order to eliminate the interference of interlayer distance expansion caused by lithium ions on the results, a control experiment was performed and the XRD test was carried out. The (002) facet of the TaS_2_ crystals shifted slightly toward lower angles after reacting with lithium atoms. However, the (002) facet of the TaS_2_ crystals shifted significantly toward lower angles after reacting with naphthyl isocyanate and octadecyl isocyanate, and the interlayer distance expanded from 6.03 Å^18^ to 10.00 Å and 11.20 Å respectively (Supplementary Fig. 2). The results showed that the expansion of TaS_2_ interlayer distance was mainly attributed to the intercalation of isocyanate molecules. In addition, the XRD results indicated that covalent modification mainly happened at the basal planes, because there is a dramatic change in (002) facet. The full width at half maximum (FWHM) of the hybrid structure changed by 0.27°, 0.74° respectively, indicating that good crystallinity was retained in the SCCM-TaS_2_ hybrid structure. Studies with cross-sectional transmission electron microscopy (TEM) also showed an increase in the interlayer distance from 6.03 Å in TaS_2_ (Fig. 2b) to 10.36 Å in the SCCM-TaS_2_ hybrid structure (Fig. 2c), which was consistent with the XRD studies (Fig. 2a). TEM images showed that the lamellar structure of SCCM-TaS_2_ crystal remained intact after covalent modification, and the single crystal structure was almost the same as that in the original.

**Fig. 3:**
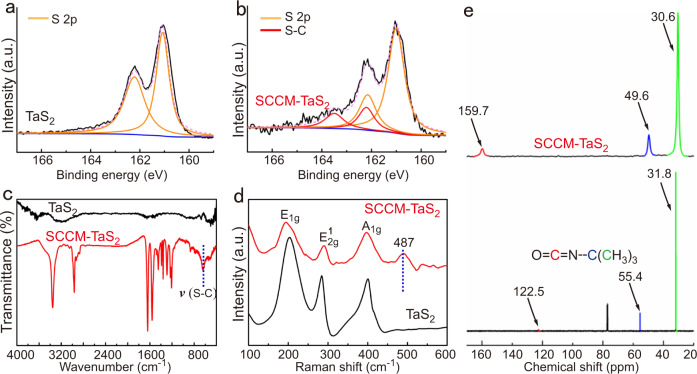
Chemical and spectral characterization from the pristine TaS_2_ and SCCM-TaS_2_ hybrid structure. XPS of S 2*p* regions from the pristine TaS_2_ single crystals (**a**) and SCCM-TaS_2_ hybrid structure (**b**). **c** FTIR spectrum of pristine TaS_2_ single crystal and SCCM-TaS_2_ hybrid structure. **d** Raman spectrum of pristine TaS_2_ single crystal and SCCM-TaS_2_ hybrid structure. **e** Liquid-state ^13^C NMR spectrum of tert-Butyl isocyanate (below) and solid-state ^13^C NMR spectrum of SCCM-TaS_2_ hybrid structure (above).

Covalent modification of the TaS_2_ crystals after reacting with tert-Butyl isocyanate was confirmed by observing signals from S 2*p* and C 1*s* regions in X-ray photoelectron spectroscopy (XPS) at ~162.0 eV and ~288.8 eV, respectively (Fig. 3a, b and Supplementary Fig. 7b). XPS analysis also revealed that S 2*p*3/2 (161.1 eV) and S 2*p*1/2 (162.4 eV) peaks were assigned to be S^2−^ in TaS_2_ crystals (Fig. 3a)^19^. In addition to the peaks of S 2*p* in Fig. 3a, two peaks were present at 163.6 eV and 162.6 eV in Fig. 3b. From XPS analysis, two peaks for the Ta 4*f* spectra have been observed, with Ta 4*f*7/2 (26.0 eV) and Ta 4*f*5/2 (28.0 eV) peaks being assigned to Ta^4+^. However, signals from the Ta 4*f* regions after covalent modification remained virtually unchanged (25–29 eV) (Supplementary Fig. 7a). Therefore, XPS analysis indicated that the covalent modification site of the side chain is on the sulfur atom^14^.

Fourier transform infrared (FTIR) spectroscopy was used to study covalent modification of the TaS_2_ crystals. The FTIR spectra of TaS_2_ crystals and the SCCM-TaS_2_ hybrid structure were shown in Fig. 3c. It can be seen that the SCCM-TaS_2_ hybrid structure displayed strong signals at 1640 cm^−1^, which were attributed to the carbonyl stretching vibration modes. The S–C stretching at 650 cm^−1^ was also clearly observed^20^. No signals from unreacted tert-Butyl isocyanate were detected, which ruled out the possibility of physical adsorption (Supplementary Fig. 4). FTIR analysis strongly suggested that the attachment of the functional groups is located on the chalcogen atoms. Figure 3d revealed the Raman spectroscopy of TaS_2_ and the SCCM-TaS_2_ hybrid structure. The Raman peaks at the 400 cm^−1^, 284 cm^−1^ and 203 cm^−1^ for the pristine TaS_2_ corresponded to the A_1g_, E_2g_^1^ and E_1g_ modes, respectively^19^. The vibration peak at 487 cm^−1^ in SCCM-TaS_2_ hybrid structure indicated the existence of S–C bonds^21^. This result was consistent with that of FTIR, which verified the reaction sites of covalent modification. These obvious lattice vibration modes suggested that the TaS_2_ crystals were highly crystalline even after modification.

To further elucidate the location of covalent modification sites on the TaS_2_ crystals, we performed solid-state ^13^C NMR spectroscopy on the SCCM-TaS_2_ hybrid structure and obtained the chemical shifts of different kinds of carbon atoms. The spectrum was referenced with an external adamantane standard in which the peak at higher chemical shift was set at 38.43 ppm. As shown in Fig. 3e, the liquid-^13^C NMR spectroscopy of tert-Butyl isocyanate showed characteristic chemical shifts (δ) corresponding to carbonyl, tertiary and methyl carbons at 122.5 ppm, 55.4 ppm and 31.8 ppm, which were referenced with chloroform-d (CDCl_3_) in which the peak at chemical shift was set at 77.0 ppm. The chemical shifts of tertiary carbon (δ 49.6 ppm) and methyl carbon (δ 30.6 ppm) in SCCM-TaS_2_ hybrid structure changed slightly compared to that of tert-Butyl isocyanate precursor. The downfield shift of carbonyl carbon (δ 159.7 ppm)^22^ compared to that in the tert-Butyl isocyanate clearly indicated the presence of different carbon-heteroatom linkage, which stemmed from covalent modification. The results indicated that carbonyl carbons of tert-Butyl isocyanate were the main attachment sites of covalent modification.

### Thermoelectric performance

**Fig. 4:**
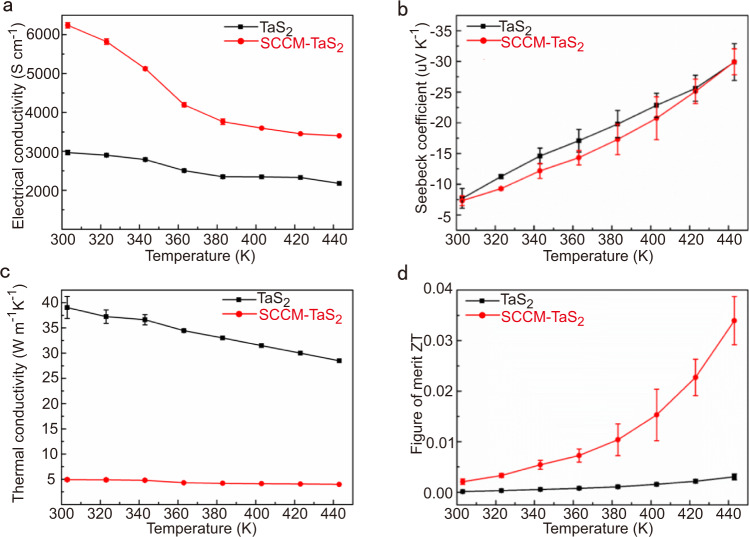
Thermoelectric properties of the pristine TaS_2_ crystals and SCCM-TaS_2_ hybrid structure. **a** Electrical conductivity. **b** Seebeck coefficient. **c** In-plane thermal conductivity. **d** In-plane thermoelectric figure of merit, ZT. All of the error bars represent the standard deviation of the results obtained from independent measurements.

Figure 4 showed the in-plane thermoelectric transport properties of 2H-TaS_2_ crystal and SCCM-TaS_2_ hybrid structure. The electrical parameters were measured by a four-probe method, which can eliminate the influence of contact resistance. The seebeck coefficient of 2H-TaS_2_ at room temperature was about −7.6 ± 1.6 uV/K, which was consistent with that reported in the literatures^23,24^. After the side chains covalent modification of the TaS_2_ crystals, the seebeck coefficient was −6.6 ± 0.3 uV/K and the electrical conductivity was 6100 S cm^−1^ at ~303 K. In order to better understand the electronic transport in TaS_2_ and SCCM-TaS_2_ hybrid structure, temperature-dependent electrical conductivity and seebeck coefficient were plotted (Fig. 4a, b). As temperature increased, the electrical conductivity reduced and the absolute value of seebeck coefficient increased. These changes indicated that the material had typical degenerate semiconductor behavior^16^.

At room temperature, the carrier concentration of TaS_2_ single crystal and SCCM-TaS_2_ hybrid structure are 1.79 × 10^22^ cm^−3^ and 5.72 × 10^22^ cm^−3^, respectively. The hall measurement showed that the carrier concentration significantly increased, which accounts well for the increase of electrical conductivity and reduction of seebeck coefficient. The in-plane mobility was calculated to be 0.67 cm^2^ V^−1^ s^−1^ in the SCCM-TaS_2_ hybrid structure, using μ = σ/(nq), where μ, σ, n and q are mobility, electrical conductivity, carrier concentration and unit electron charge, respectively. The decrease of mobility from 1.40 cm^2^ V^−1^ s^−1^ for the TaS_2_ single crystal to 0.67 cm^2^ V^−1^ s^−1^ for the SCCM-TaS_2_ is due to the interaction between organic molecules and inorganic layers and the scattering of electrons at many interfaces^25^.

Experimental measurement of thermal conductivity of 2D TMDs materials is still a challenging task. In this experiment, the thermal conductivity was measured with suspended micro bridge electrodes for small samples, which can eliminate the influence of substrate heat conduction^26–28^. The in-plane thermal conductivity of the TaS_2_ crystal and SCCM-TaS_2_ hybrid structure were measured to be 28.5 W m^−1^ K^−1^ and 4.0 W m^−1^ K^−1^ at 443 K, respectively (Fig. 4c). Phonons and electrons collectively contributing to the thermal conductivity, after minus the electronic thermal conductivity estimated based on the Wiedemann-Franz law (κ_e_ = σLT)^29^, the in-plane lattice thermal conductivity of the TaS_2_ crystal and SCCM-TaS_2_ hybrid structure were estimated to be 36 W m^−1^ K^−1^ and 0.6 W m^−1^ K^−1^ at 303 K, respectively. Due to the significant decrease of thermal conductivity, the in-plane ZT value of SCCM-TaS_2_ hybrid structure is 10 times that of TaS_2_ crystal, reaching ~0.04 at ~443 K.

The decrease of lattice thermal conductivity is due to the introduction of organic side chains through covalent bonds, which leads to the enhancement of phonon scattering. The covalent bonds between inorganic layer and organic layer are much stronger than van der Waals interaction. It is speculated that covalent bonds are more effective than van der Waals interaction in enhancing phonon scattering and reducing lattice thermal conductivity^30^. In addition, the thermal conductivity decreased slightly with the increase of temperature (Fig. 4c). This is because when the thermal conductivity is dominated by anharmonic phonon-phonon interaction, the mean free path of phonon decreases with the increase of temperature^31^.

As the organic side chains were directly bonded to TMDs materials by covalent modification, the covalent bonds are stronger. Therefore, the products achieved by this method have higher thermal stability than that of the organic guest intercalated compounds by van der Waals^16^. We studied the thermal stability of the hybrid structure by thermogravimetric analysis (TGA). The traces of the first derivatives of the TGA traces showed that the decompositions of the functional groups occurred at 204 °C for SCCM-TaS_2_ hybrid structure, which indicated the SCCM-TaS_2_ hybrid structure had good thermal stability. TGA revealed that the degree of modification was up to 22.5 at.% relative to the TaS_2_ which has been calculated by measuring the weight loss at 400 °C (Supplementary Fig. 3).

## Discussion

In summary, we have successfully developed an effective strategy for organic side chains covalent modification of TMDs single crystal. The organic groups were covalently bonded to TMDs single crystal directly to form the side chains covalent modification hybrid structure with superior crystallinity. After covalent modification, the thermal conductivity was reduced to one-seventh of the TaS_2_ crystal, and the ZT value had been 10 times in the SCCM-TaS_2_ hybrid structure than that in TaS_2_ crystal. Tests showed the covalent hybrid structure had good thermal stability due to the existence of robust covalent bonds. This strategy of organic covalent modification can be extended to other layered materials and offers attractive platforms to obtain high ZT value thermoelectric devices.

## Methods

### Preparation of 2H-TaS_2_ single crystal

2H-TaS_2_ was synthesized by heating stoichiometric ratios elements of Ta (99.99%, Alfa Aesar) and S (99.999%, Alfa Aesar) in an evacuated quartz tube at 900 °C for several days, followed by slowly cooling the quartz tube and its contents. The single crystal of 2H-TaS_2_ was grown by the standard chemical vapor transport method.

### Preparation of SCCM-TaS_2_

The TaS_2_ single crystal and lithium foil were used as the cathode and anode in electrochemical reaction cells, the self-prepared solution was used as the electrolyte. As shown in Fig. 1, in the first step, the TaS_2_ single crystal was charged by electrochemical reduction. In the second step, the electrochemical reduction products were immersed in tert-butyl isocyanate (98%), naphthyl isocyanate (99%), and octadecyl isocyanate (98%) for one week at room temperature. The products from these reactions were thoroughly washed with acetone before analysis.

### Fabrication of devices

The nanosheets with the thickness of about 100 nm were obtained by mechanical exfoliation, and then transferred to the substrates or suspended microelectrodes. 100 nm of Au was deposited on the top of samples as electrodes with a shadow mask by vacuum thermal evaporation at a rate of about 0.3 Å s^−1^. More details are described in the Supplementary Information.

### Characterization

X-ray diffraction XRD was performed on a Panalytical-Empyrean. HR-TEM (JEOL JEM-2100F) was employed to image the samples. XPS was conducted using an ESCALab250-Xi electron spectrometer from VG Scientific with 300 W Al Kα radiation. The IR spectroscopy was performed on a Bruker-Tensor-27. Raman was performed with a 532 nm laser under ambient conditions (in Via-Reflex). The solid-state NMR experiments were performed on a Bruker 400 MHz and the samples were carefully packed into a 3.2 mm zirconia rotor. Thermally evaporated 100 nm Au films were used as metal electrodes for electrical conductivity and seebeck coefficient measurement. Thermogravimetric analysis (TGA) was performed on a NETZSCH-STA 409 PC. TG analyzer equipped with thermal analysis controller. More details are described in the Supplementary Information.

### Reporting summary

Further information on research design is available in the Nature Research Reporting Summary linked to this article.

## Supplementary information

Supplementary Information

Reporting Summary

Lasing Reporting Summary

## Data Availability

The authors declare that the experimental data supporting the results of this study can be found in the paper and its Supplementary Information file. The detailed data for the study is available from the corresponding author upon request.
