# Supplementary material for: Organic covalent modification to improve thermoelectric properties of TaS2
Source: Nat Commun. 2022 Jul 29;13:4401. doi: 10.1038/s41467-022-32058-w (PMC9338255; doi:10.1038/s41467-022-32058-w)
Supplement: Supplementary file 1 — Supplementary Information [file 41467_2022_32058_MOESM1_ESM.pdf]

Supplementary Information

Organic covalent modification to improve thermoelectric properties

Shaozhi Wang[1,3,†], Xiao Yang[2,3,†], Lingxiang Hou[1,3], Xueping Cui[1*], Xinghua Zheng[2,3*], Jian Zheng[1*]

[1]Beijing National Laboratory for Molecular Sciences, Key Laboratory of Organic Solids, Institute of Chemistry, Chinese Academy of Sciences, Beijing 100190, China.

[2]Institute of Engineering Thermophysics, Chinese Academy of Sciences, Beijing 100190, China.

[3]University of Chinese Academy of Sciences, Beijing 100049, China.

[†]These authors contributed equally: Shaozhi Wang, Xiao Yang.

Correspondence and requests for materials should be addressed to J.Z. (zhengjian@iccas.ac.cn)

This PDF file includes:

Supplementary Methods

Supplementary Characterization

Supplementary Methods

Preparation of TaS_2 bulk single crystal:

2H-TaS_2 was synthesized by heating stoichiometric ratios elements of Ta (99.99%, Alfa Aesar) and S (99.999%, Alfa Aesar) in an evacuated quartz tube at 900°C for several days, followed by slowly cooling the reaction quartz tube and its contents. The single crystal of 2H-TaS_2 were grown by the standard chemical vapor transport method.

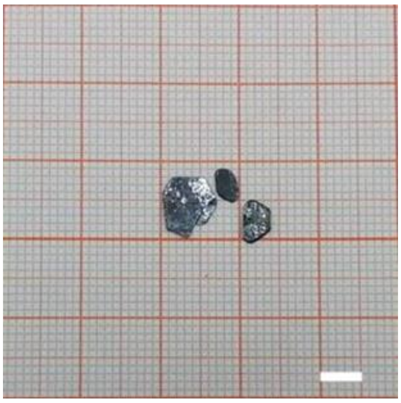

Supplementary Figure 1. Photograph of the 2H-TaS_2 bulk single crystal. (Scale bars: 5 mm)

Supplementary Characterization

XRD patterns of TaS_2 and TaS_2 modified with different organic molecules:

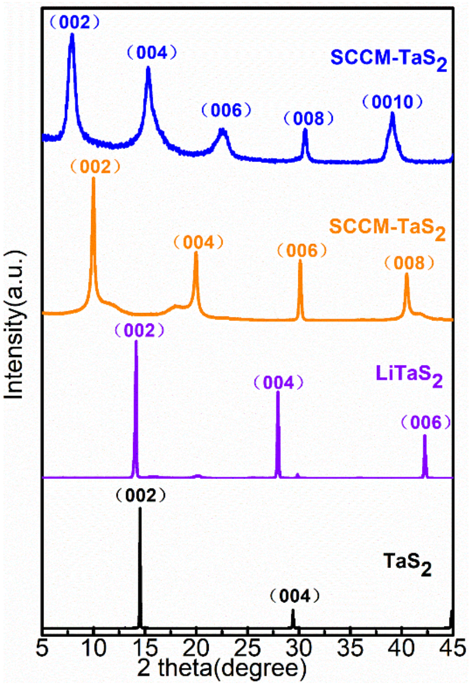

Supplementary Figure 2. XRD patterns of TaS_2 bulk single crystals, $LiTaS_2$, SCCM-TaS_2 hybrid structure with naphthyl isocyanate (orange) and with octadecyl isocyanate (blue).

TGA measurements on the pristine TaS_2 and SCCM-TaS_2 hybrid structure：

After purging the system for 30 min at 40 ^oC, samples were heated under nitrogen at 10 ^oC / min from 40 ^oC up to 500 ^oC. The weight loss has been recorded for pristine TaS_2 and the SCCM-TaS_2 hybrid structure (Supplementary Figure 3). The derivative signal from pristine TaS_2 sample remains relatively flat. At the opposite, the derivative of the weight loss shows peak at 204 ^oC for the degradation of the SCCM-TaS_2 hybrid structure.

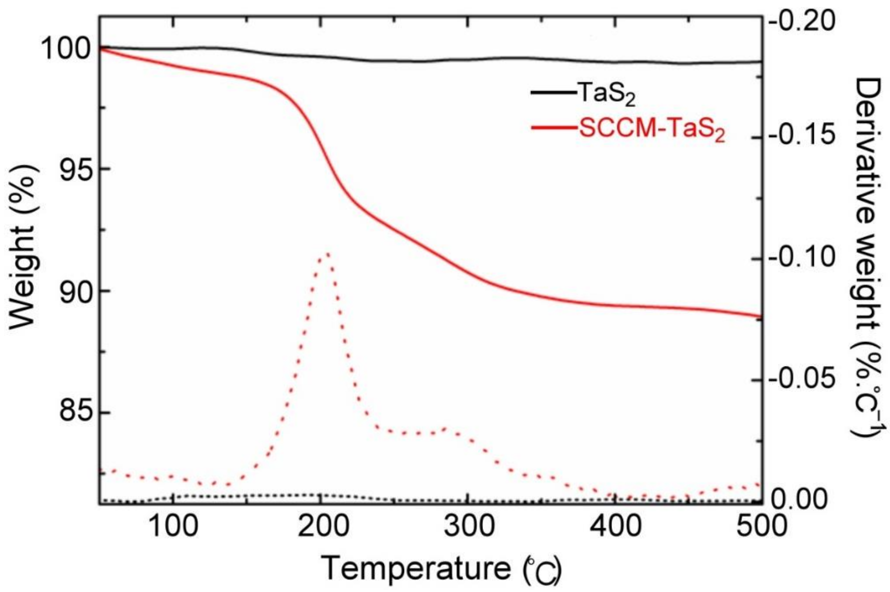

Supplementary Figure 3. Thermogravimetric analysis (TGA) of the pristine TaS_2 and SCCM-TaS_2 hybrid structure up to 500 ^oC under nitrogen atmosphere.

IR spectroscopy of tert-Butyl isocyanate and SCCM-TaS_2 hybrid structure:

The IR spectra of tert-Butyl isocyanate and SCCM-TaS_2 hybrid structure bring the first evidence of covalent modification of the functional groups. We can preliminarily determine the reaction sites of the reactants from the IR spectra. The stretching vibration of (-N=C=O) band in 2280 cm^{-1}-2200 cm^{-1} is very strong in tert-Butyl isocyanate. Compared with the IR spectra of tert-Butyl isocyanate, the peak (-N=C=O) disappeared in the SCCM-TaS_2 hybrid structure, indicating that the chemical bond (-N=C=O) was broken. The S-C stretching is identified at ~ 650 cm^{-1} in SCCM-TaS_2 hybrid structure. It is confirmed that the organic covalent modification site is on the carbon atoms of N = C = O functional group.

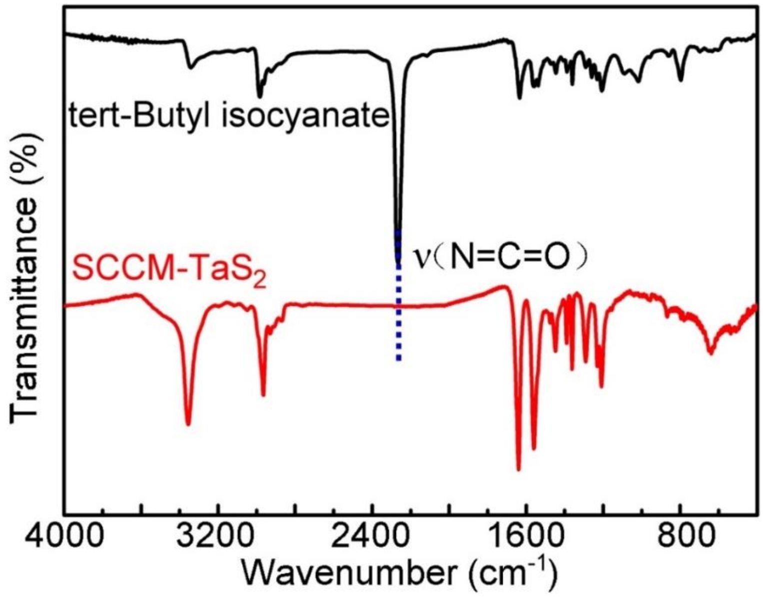

Supplementary Figure 4. IR spectra of tert-Butyl isocyanate (black) and SCCM-TaS_2 hybrid structure (red).

Nuclear Magnetic Resonance spectroscopy (NMR):

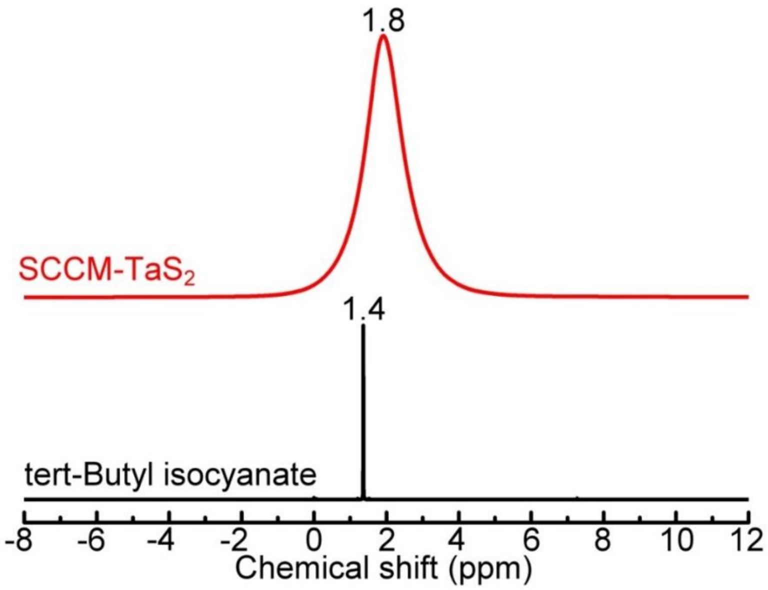

Supplementary Figure 5. Liquid-state ^{1}H NMR spectra of tert-Butyl isocyanate (black), Solid-state ^{1}H NMR spectra of SCCM-TaS_2 hybrid structure (red).

X-ray photoelectron spectroscopy (XPS) on the pristine TaS_2 and SCCM-TaS_2 hybrid structure:

There is no Li peak in Supplementary Figure 6, which can eliminate the interference of Li ions on the performance test. XPS spectra for the Ta 4*f* and C 1*s* regions were shown in Supplementary Figure 7. The presence of functional groups can be seen on the C 1*s* regions (288-290 eV).

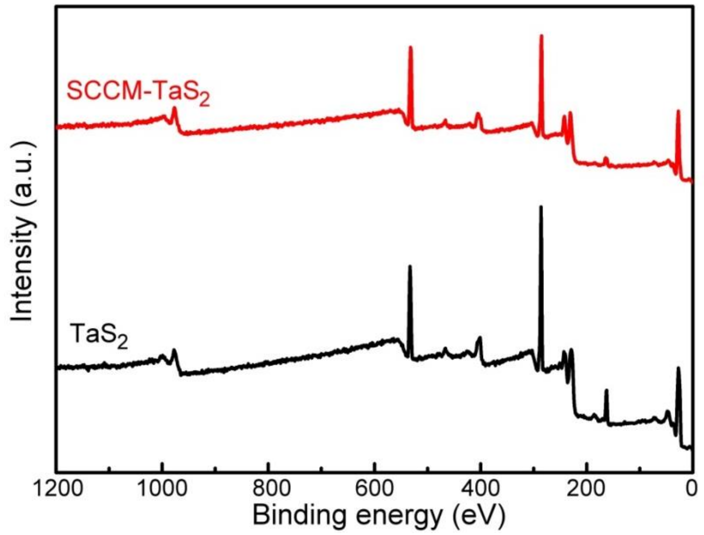

Supplementary Figure 6. XPS survey spectra of the pristine TaS_2 and SCCM-TaS_2 hybrid structure.

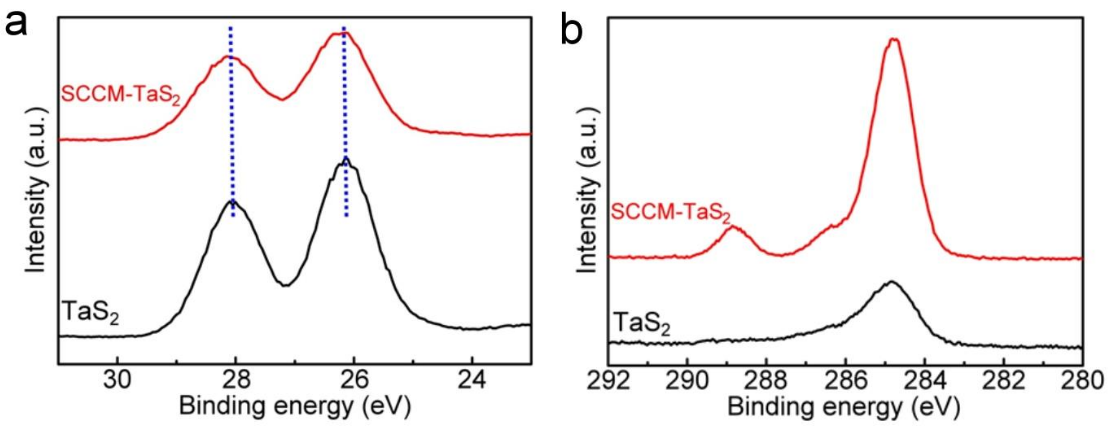

Supplementary Figure 7. XPS spectra of Ta 4*f* (a) and C 1*s* (b) regions from the pristine TaS_2 and SCCM-TaS_2 hybrid structure.

Electrical conductivity and Seebeck coefficient measurement:

The electrical conductivity was measured in vacuum condition based on the four-probe method to exclude the effects of electrical contact resistance. Before the measurement, the sample was mechanically exfoliated into thin sheets (d~100 nm) and transferred to substrates. Parallel gold (d~100 nm) was evaporated as the electrode. The apparatus is a standard system for the measurement of Seebeck coefficient. The apparatus we used include Agilent B 1500A and a vacuum chamber. The thermal voltage was measured by Agilent B 1500A in vacuum.

Hall measurement system:

The carrier concentration and mobility were measured in vacuum condition based on the Van der Pauw method. The measurement was carried out using the commercial PPMS measurement system (Quantum Design Inc. PPMS DynaCool-9T).

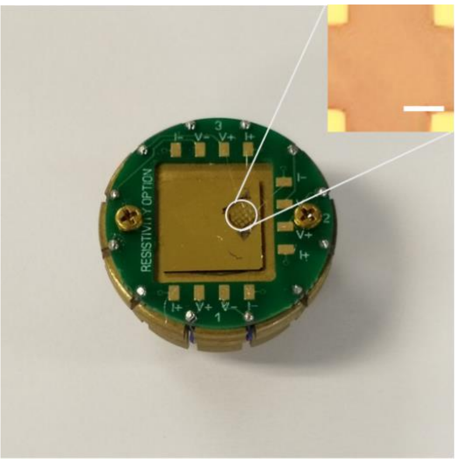

Supplementary Figure 8. Schematic illustration of the experimental setup for the Hall measurement. Sample configuration for electrical conductivity and carrier concentration measurement using the Van der Pauw method. Gold with a thickness of 100 nm was deposited as electrodes with a shadow mask by vacuum thermal evaporation. The four corners of the sample are connected to the electrodes of the DC electrical sample holder through welding wires. inset: the optical microscopic image of mechanically exfoliated nanosheets on substrate; Scale bars:35μm.

Fabrication of suspended microelectrodes:

The electrodes were fabricated by standard electron-beam lithography which provided by the Institute of Engineering Thermophysics, Chinese Academy of Sciences. In order to obtain the in-plane thermal conductivity of the pristine TaS_2 and SCCM-TaS_2 hybrid structure, in this measurement, the sample was transferred to the suspended microelectrodes by PMMA, and tested in vacuum.

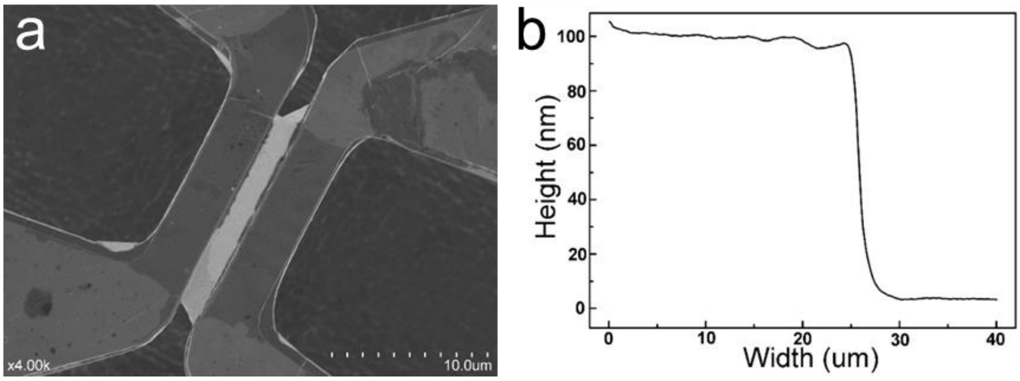

Supplementary Figure 9. SEM image of sample on the suspended microelectrodes (a) and the thickness of mechanically exfoliated samples (b).

Thermal conductivity measurement:

Measurement system included lock-in amplifier 1, preamplifier 2, differential amplifier 3, adjustable resistor 4 and measuring terminal 5. The measuring method is conducted by using microelectrode #1 for heating and microelectrode #2 for measuring the temperature rise of the heat conduction caused by the heat flow through the sample. Microelectrode #1 is connected to a lock-in amplifier, and this lock-in amplifier is used to heat the microelectrode and measure the temperature rise of microelectrode #1. Microelectrode #2 is also connected to the other lock-in amplifier to measure the temperature rise of the heat receiving end. Using AC power at the same frequency can reduce the environmental interference. This measurement system has been calibrated by a standard platinum wire with a diameter of 20 μm placed on the suspended electrode, and its uncertainty is about 3.2%. This measurement process was carried out in room temperature and high vacuum environment (less than10^{-4} Pa).

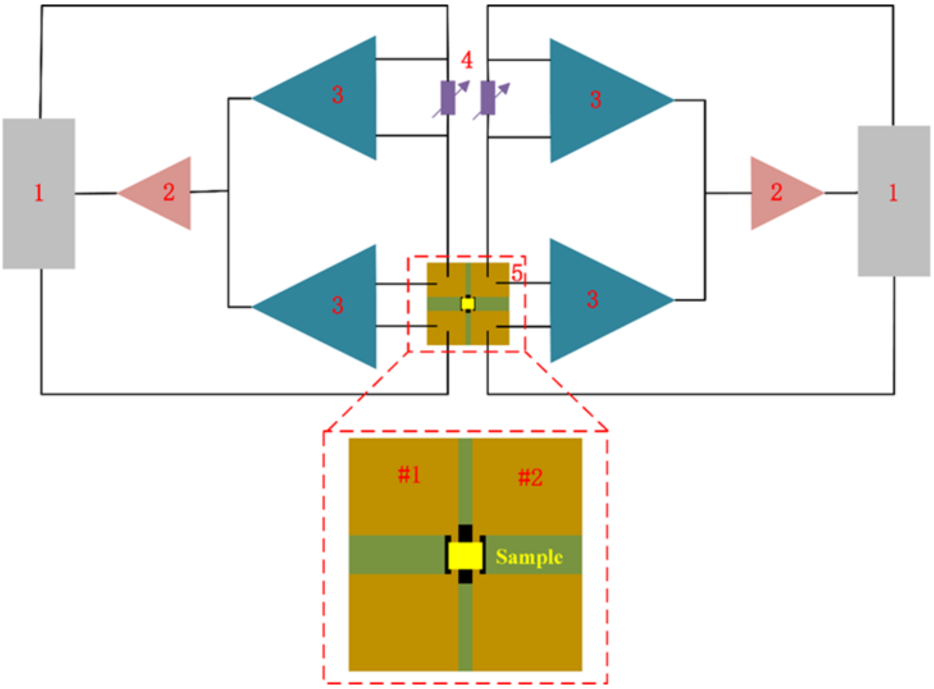

Supplementary Figure 10. The measurement system of thermal conductivity.
